# Spatial Feature Structure Generation Network for Airborne LiDAR Point Cloud Semantic Segmentation

**DOI:** 10.3390/s26102996

**Published:** 2026-05-09

**Authors:** Ting Guo, Zeyu Tian, Xinqi Liu, Bin Leng, Gangwei Mi

**Affiliations:** 1 College of Mechanical and Electrical Engineering, Heilongjiang Institute of Technology, Harbin 150050, China; guoting@hljit.edu.cn; 2 College of Surveying and Mapping, Heilongjiang Institute of Technology, Harbin 150050, China; liuxinqi@hljit.edu.cn (X.L.); lengbin@hljit.edu.cn (B.L.); migangwei@hljit.edu.cn (G.M.); 3 Xi’an Research Institute of Surveying and Mapping, Xi’an 710054, China; 4 State Key Laboratory of Spatial Datum, Xi’an 710054, China

**Keywords:** semantic segmentation, attribute feature, coordinate feature, spatial structure feature, feature fusion

## Abstract

With the rapid development of airborne Light Detection and Ranging (LiDAR) sensors, point clouds have been widely applied in fields such as surveying and mapping engineering, intelligent monitoring, and autonomous driving. However, the irregular structure of point clouds seriously affects the segmentation, recognition, and understanding of point clouds. To address the challenge, this paper proposes a spatial feature structure generation network (SFSGNet). The spatial feature structure generation (SFSG) module can deeply fuse attribute features and spatial coordinate features to generate highly expressive spatial structure features in both semantic and spatial contexts, and through the spatial structure features, SFSGNet can effectively handle irregular point clouds, describe the structure of point cloud objects, and achieve accurate point cloud semantic segmentation. We evaluate our SFSGNet using the ISPRS 3D Labeling Benchmark dataset, DALES dataset and OpenGF dataset. Experimental results show that our SFSGNet achieves an average F1 score 74.4% and overall accuracy 85.4% on the ISPRS dataset, achieves an average F1 score 89.3% and overall accuracy 98.1% on the DALES dataset, and achieves an average F1 score 98.2% and overall accuracy 98.5% on the OpenGF dataset. Compared with other representative models, our SFSGNet demonstrates excellent segmentation performance and strong generalization.

## 1. Introduction

Point cloud semantic segmentation aims to assign a semantic label to each point, converting raw 3D data into actionable information. It serves as a foundation for advanced applications such as autonomous driving, robotics navigation, infrastructure inspection, and environmental monitoring. Early semantic segmentation methods for point clouds primarily relied on traditional machine learning approaches, which heavily depended on manually crafted features such as geometric attributes (e.g., normal vectors, curvature) [1] and contextual relationships. Methods like conditional random fields (CRFs) and support vector machines (SVMs) [2] were commonly employed but were limited by their reliance on hand-crafted features and inability to capture complex, high-level semantic information. With the rise of deep learning, point cloud processing has evolved significantly.

### 1.1. Deep-Learning-Based Point Cloud Processing Methods

PointNet [3] directly processes raw point clouds through a permutation-invariant neural network architecture, providing a unified and efficient framework for tasks like object classification and segmentation. PointNet++ [4] directly processes raw point clouds by recursively applying PointNet on nested partitions of the input set, leveraging metric space distances to hierarchically learn multi-scale local features. KPConv [5] directly processes raw point clouds by applying convolutional weights through learnable kernel points in Euclidean space, utilizing deformable convolutions to adapt to local geometry without any intermediate representation. PointCNN [6] processes raw point clouds by learning an X-transformation that weights input features and permutes points into a latent canonical order, generalizing standard CNNs for effective feature learning.

Rahmanti et al. [7] directly utilize raw 3D LiDAR point cloud data by converting it into PCD format. This data serves as input for a deep learning algorithm, such as a convolutional neural network. PAConv [8] generates convolutional kernels adapted to the spatial positions of point clouds. This ensures diversity in the generated kernels. It effectively addresses the sparsity and irregularity of point clouds. It also overcomes the high computational cost and limited flexibility of traditional methods. RS-CNN [9] extends CNNs from regular grids to irregular point cloud scenarios. It builds spherical neighborhoods using sampled points as centroids. This enables more discriminative shape-aware learning. The proposed EPN-GPRFM [10] directly processes raw 3D point clouds by cyclically mining features from discarded points and enhancing prototype representation using query samples within a pure 3D framework for few-shot learning. Sparse LiDAR processes point clouds [11] by enhancing the PointNet++ network with a point cloud structure-adaptive module and a global feature encoding module to improve object classification. LGENet [12] is based on the KPConv network. It designs hybrid 2D-3D convolution blocks. It introduces segment-based edge-conditioned convolution and a channel attention module. These components improve overall accuracy for point cloud semantic segmentation. They also enhance robustness across different categories. SPG [13] achieves high-precision end-to-end semantic segmentation for 3D point clouds. It incorporates an improved lightweight edge-conditioned convolution. Labels are mapped back to the original point cloud. This yields accurate and fast segmentation results. The RSSP framework [14] directly processes LiDAR point clouds as sparse tensors, utilizing novel sparse strided operations to achieve real-time 3D scene understanding at high resolutions. DG-Net [15] builds a DNFC module to fuse local geometric structures and semantic features. It captures discriminative local contextual features. This enhances the local feature representation ability of point clouds. It also improves segmentation accuracy for small objects and boundaries. PointCutMix [16] designs two strategies: random point replacement and centroid-based neighbor point replacement. These strategies fuse point clouds to generate new training samples. This significantly improves segmentation performance for rare categories in point clouds.

1D-FCN [17] achieves end-to-end semantic annotation for point clouds. It learns point-level local features and block-level global contextual features through permutation-invariant max pooling layers. This approach eliminates the need for manual feature engineering. It also significantly improves inference efficiency. DGCNN [18] enables efficient point cloud learning with dynamic graph updating and the EdgeConv module. It fuses global–local features of points and their neighborhoods via the EdgeConv module, thus achieving efficient extraction of geometric and semantic features from irregular point cloud data. PointSSM [19] realizes large-scale point cloud semantic segmentation based on state space models. It combines Mamba with convolution complementarily and adopts bidirectional modeling, thus achieving efficient global modeling on large-scale point clouds. GeneCGAN [20] achieves unsupervised reconstruction of specific 3D points. It injects global features as conditional information through prior fusion operations, thus significantly reducing reconstruction errors and realizing uniform distribution of generated point clouds.

MS-PCNN [21] improves the PointCONV operator to enable direct point-wise convolution on 3D point clouds. It designs a U-shaped sampling architecture to fuse multi-scale global and local features. This achieves efficient high-level feature extraction for unordered and non-uniformly dense 3D point clouds.

In addition to mainstream deep learning architectures, Liu et al. [22] explored advanced lightweight tuning strategies for RGB + X multi-modal semantic segmentation. They proposed a cross-prompt adapter equipped with low-rank adaptation (LoRA) to unlock the potential of pre-trained foundation models in both RGB and non-RGB modalities, achieving state-of-the-art performance.

### 1.2. Attention-Mechanism-Based Point Cloud Processing Methods

MASNet [23] designs a stacked pillar mixed attention module to enhance the pillar feature extraction capability. It adopts an end-to-end one-stage architecture of pillarization feature and extraction feature aggregation, converts 3D point clouds into 2D pillar structures, and then processes them through the above module to output 3D bounding boxes. 3D-ARSS [24] uses two lightweight modules: spatial attention and channel attention. It combines sparse tensors for efficient computation across the network. This enables real-time LiDAR point cloud segmentation on edge devices. The method balances segmentation accuracy with computational efficiency. PCT [25] designs three modules: coordinate-based input embedding, offset attention, and neighbor embedding. It achieves invariance in point cloud processing. It also captures both global context and local geometric information. SASENet [26] designs a semantic space enhancement module. It enriches point cloud semantics via infrared image semantic segmentation, embeds the CBAM attention module and Bidirectional Feature Interaction Module (BFIM), and achieves cross-modal bidirectional feature fusion of point clouds and infrared images. DGACN [27] enables simultaneous learning of low-level extrinsic and high-level intrinsic features for point cloud classification. It adopts a dual graph attention mechanism, error feedback fusion and hierarchical feature extraction, thus achieving strong robustness to real-scene issues such as background interference and object incompleteness. HAPGN [28] enables efficient extraction of local fine-grained and global hierarchical features for point cloud classification. With a Gated Graph Attention Network (GGAN) and a Hierarchical Graph Pooling Module (HiGPool) as the core, it achieves strong tolerance to low-density point clouds and Gaussian noise. PAN [29] captures both local geometric features and long-range spatial correlations to achieve accurate point cloud semantic segmentation. It adopts a multi-directional neighborhood search and edge-attention-weighted aggregation, thus enabling precise differentiation of homogeneous objects in sparse and irregular point clouds. AGNet [30] improves the accuracy of point cloud classification and segmentation. It adopts k-NN local graph construction, attention pooling for adaptive feature selection, and multi-scale feature fusion, thus enabling efficient extraction of key features in sparse and irregular point cloud scenarios. Att-AdaptNet [31] efficiently captures local geometric features and global key information of point clouds. It adopts adaptive kernel functions, global attention mask enhancement, and dual-pooling for multi-dimensional feature aggregation, thus maintaining high classification accuracy even in occluded point cloud scenarios. MFNet [32] fully captures local, point-set and multi-scale features of point clouds. It adopts hierarchical extraction of multi-scale features and channel-attention-weighted fusion, thus effectively distinguishing semantic objects with similar structures in complex point cloud scenarios. SE-PointNet++ [33] enables accurate classification of multispectral LiDAR point clouds. It adopts multispectral data fusion, SE attention channel weighting and multi-scale feature extraction, thus effectively distinguishing easily confused categories in point cloud scenarios with uneven density. DCTN [34] achieves simultaneous improvement in accuracy and efficiency for point cloud classification. It integrates convolution with a Transformer and adopts hierarchical feature extraction and multi-scale neighborhood aggregation, thus enabling efficient extraction of discriminative features in irregularly distributed point cloud scenarios.

## 2. Methods

**Figure 1:**
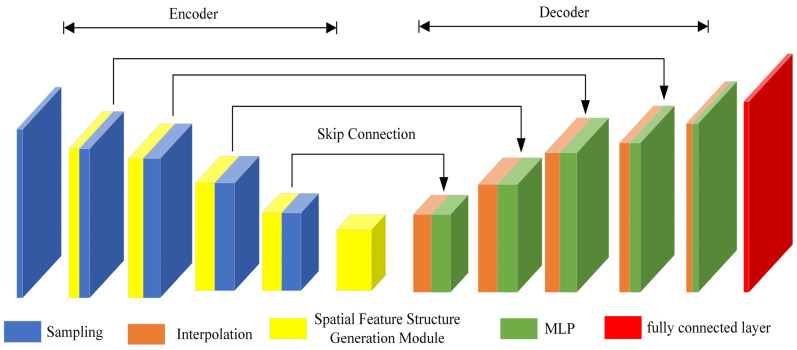
Spatial feature structure generation network.

This chapter presents the proposed spatial feature structure generation network (SFSGNet) for airborne LiDAR point cloud semantic segmentation, as shown in Figure 1. Unlike conventional approaches that either project 3D point clouds onto 2D planes (causing information loss) or struggle with irregular data structures, SFSGNet adopts a hierarchical encoder–decoder architecture for end-to-end feature learning and classification. Its core innovation lies in the spatial feature structure generation (SFSG) module, which jointly models geometric coordinates and attribute features via a multi-path architecture to progressively expand and fuse multi-scale information. This design preserves fine-grained spatial structures, enhances semantic expressiveness, and resolves key limitations such as neighborhood ambiguity and insufficient interaction between spatial and semantic features. By fully exploiting the complementary information between spatial coordinates and attribute features, the network generates discriminative representations for robust structure description and accurate semantic labeling in complex scenes. The SFSG module is detailed in Section 2.1, followed by the full network architecture in Section 2.2.

### 2.1. Spatial Feature Structure Generation (SFSG) Module

Effective integration of geometric coordinate features and attribute features is critical to learning discriminative representations for point cloud semantic segmentation. To fully exploit the complementary value of spatial location information and attribute information, we propose the spatial feature structure generation (SFSG) module. This module performs hierarchical expansion and deep interaction of coordinate and attribute features and constructs two parallel fusion branches to simultaneously capture high-level semantic context and retain fine-grained geometric details. Through hierarchical aggregation of the dual-path features, the module generates highly expressive spatial structure features, which strengthens the modeling capability of irregular point cloud structures and enhances the discrimination and structural awareness of the learned feature representations.

**Figure 2:**
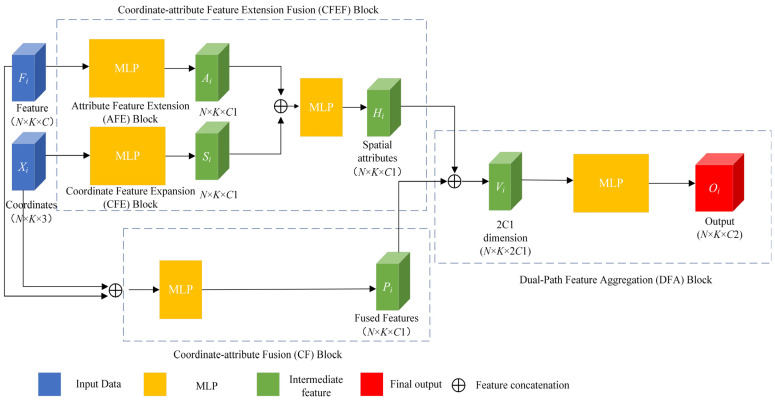
Spatial feature structure generation module.

The point cloud is input into the ball query [4] method around a set of center points to generate the local neighborhood $P=\{P_{1},P_{2},\ldots ,P_{N}\}\;\epsilon \;R^{K\times N\times (3+C)}$ of the point cloud. N is the number of center points, K is the number of neighborhood points, and C is the number of attribute features. The local neighborhood P of the point cloud includes the attribute feature $F\;\epsilon \;R^{K\times N\times C}$ and coordinate feature $X\;\epsilon \;R^{K\times N\times 3}$. The SFSG module utilizes these coordinate and attribute features to generate spatial structure features through five sub-blocks: attribute feature extension block, coordinate feature expansion block, coordinate–attribute feature extension fusion block, coordinate–attribute fusion block, and dual-path feature aggregation block. These sub-blocks implement the aforementioned dual-path fusion strategy. The SFSG module is shown in Figure 2.

#### 2.1.1. Attribute Feature Extension (AFE) Block

In the attribute feature extension block, the attribute feature of each point is input into a shared Multi-Layer Perceptron (MLP). This operation maps the attribute feature F into a higher-dimensional feature space with C1 channels to produce the expanded attribute feature of each point. The initial attribute of each point is the reflectance intensity. This map can be formulated as follows:

(1) $$A_{i}=\sigma \times (\sum_{i=1}^{n}W_{2}^{\left(a\right)}\times (\sum_{i=1}^{n}W_{1}^{\left(a\right)}\times F_{i}+b_{1}^{a})+b_{2}^{a})$$

where $A_{i}\;\epsilon \;R^{C_{1}}$ represents the output feature of point *i* after attribute feature expansion; $F_{i}\;\epsilon \;R^{C}$ represents the input feature of point *i*; $W_{1}^{\left(a\right)}\;\epsilon \;R^{C1\times C}$ and $W_{2}^{\left(a\right)}\;\epsilon \;R^{C1\times C1}$ represent the weight matrices of the first and second layers of the MLP; $b_{1}^{a}\;\epsilon \;R^{C_{1}}$ and $b_{2}^{a}\;\epsilon \;R^{C_{1}}$ represent the corresponding bias vectors; and σ(·) represents the nonlinear activation function (ReLU).

The attribute feature extension block can encode more complex patterns and provide a richer and more discriminative representation for subsequent processing stages.

#### 2.1.2. Coordinate Feature Expansion (CFE) Block

In the coordinate feature expansion block, the raw three-dimensional coordinates (x,y,z) of each point are fed into a shared Multi-Layer Perceptron (MLP). This MLP transforms the low-dimensional coordinate into a high-dimensional feature space with C1 channels to produce the expanded coordinate feature of each point. The above operation can be formulated as follows:

(2) $$X_{i}=(x_{i},y_{i},z_{i})\;\epsilon \;R^{3}$$

(3) $$S_{i}=\sigma \times (\sum_{i=1}^{n}W_{4}^{\left(a\right)}\times (\sum_{i=1}^{n}W_{3}^{\left(a\right)}\times X_{i}+b_{1}^{a})+b_{2}^{a})$$

where $X_{i}$ represents the three-dimensional coordinate of point *i*; $S_{i}$ represents the expanded coordinate feature; $W_{3}^{\left(a\right)}\;\epsilon \;R^{C1\times C}$ and $W_{4}^{\left(a\right)}\;\epsilon \;R^{C1\times C1}$ represent the weight matrices of the first and second layers of the MLP; $b_{1}^{a}\;\epsilon \;R^{C_{1}}$ and $b_{2}^{a}\;\epsilon \;R^{C_{1}}$ represent the corresponding bias vectors; and σ(·) represents the nonlinear activation function (ReLU).

The coordinate feature expansion block can encode the location context and provide a rich spatial representation. This block can explicitly capture the geometric structure and local spatial relationships of the point cloud.

#### 2.1.3. Coordinate–Attribute Feature Extension Fusion (CFEF) Block

This coordinate–attribute feature extension fusion block concatenates the expanded attribute feature with the expanded coordinate feature and feeds the combined representation into an additional MLP. The C1-dimensional expanded attribute feature obtained by the attribute feature extension block is concatenated with the C1-dimensional expanded coordinate feature obtained by expanding the coordinates to form a 2 × C1-dimensional concatenated feature representation. This concatenated feature is then fed into another MLP to output a more expressive C1-dimensional feature, which is referred to as the spatial-attribute feature. The above operation can be formulated as follows:

(4) $$U_{i}=[A_{i}⊕S_{i}]\;\epsilon \;R^{2\times C1}$$

(5) $$H_{i}=\sigma \times (\sum_{i=1}^{n}W_{6}^{\left(a\right)}\times (\sum_{i=1}^{n}W_{5}^{\left(a\right)}\times U_{i}+b_{1}^{a})+b_{2}^{a})\;\epsilon \;R^{C1}$$

where $U_{i}$ represents the concatenated feature; $A_{i}$ represents the expanded coordinate feature; $S_{i}$ represents the expanded coordinate feature from the coordinate feature expansion block; ⊕ represents the feature concatenation operation; $W_{5}\;\epsilon \;R^{C1\times 2C1}$ and $b\;\epsilon \;R^{C1}$ represent the weight matrix and bias of the fusion MLP; and $H_{i}$ represents the spatial-attribute feature.

The coordinate-attribute feature extension fusion block can achieve the interaction between the attribute feature and coordinate feature to generate a more expressive spatial-attribute feature representation.

#### 2.1.4. Coordinate–Attribute Fusion (CF) Block

The coordinate–attribute fusion block directly concatenates the raw three-dimensional coordinates (x,y,z) of each point with the corresponding attribute feature and feeds the concatenated feature into the MLP. This block enables the network to simultaneously leverage the spatial coordinate and attribute information from a joint perspective to produce a C1-dimensional fusion feature. The above operation can be formulated as follows:

(6) $$P_{i}=\sigma \times (\sum_{i=1}^{n}W_{8}^{\left(a\right)}\times (\sum_{i=1}^{n}W_{7}^{\left(a\right)}\times [X_{i}⊕F_{i}]+b_{1}^{a})+b_{2}^{a})\;\epsilon \;R^{C1}$$

where $X_{i}$ represents the raw coordinates; $F_{i}$ represents the raw attribute (intensity). $P_{i}$ represents the fusion feature; and $W_{7}\;\epsilon \;R^{C1\times (3+C)}$ and $b\;\epsilon \;R^{C1}$ represent the weights and biases of the parallel-branch MLP.

#### 2.1.5. Dual-Path Feature Aggregation (DFA) Block

The spatial-attribute feature $H_{i}$ obtained from the coordinate–attribute feature extension fusion block and the fusion features $P_{i}$ produced by the coordinate–attribute fusion block are concatenated to form a 2 × C1-dimensional aggregation feature $V_{i}$. This aggregation feature is subsequently fed into a shared MLP to output the final C2-dimensional spatial structure feature $O_{i}$. The above operation can be formulated as follows:

(7) $$V_{i}=[H_{i}⊕P_{i}]\;\epsilon \;R^{2\times C1}$$

(8) $$O_{i}=\sigma \times (W_{7}\times V_{i}+b_{6})\;\epsilon \;R^{C2}$$

where $V_{i}$ represents the aggregation feature; $W_{6}\;\epsilon \;R^{C2\times 2C1}$ and $b_{4}\;\epsilon \;R^{C1}$ represent the weight matrix and bias of the output MLP; and $O_{i}$ represents the spatial structure feature.

The spatial structure feature $O_{i}$ is then input into the max pooling operation.

By this hierarchical concatenation and fusion mechanism, the dual-path feature aggregation block can effectively aggregate information from two paths to generate highly expressive spatial structure features in both semantic and spatial contexts.

### 2.2. Spatial Feature Structure Generation Network

The encoder part of the spatial feature structure generation network (SFSGNet) adopts a hierarchical structure. By performing layer-by-layer sampling and a spatial feature structure generation (SFSG) module, the encoder can gradually extract multi-scale feature representations of point clouds. The network takes the local neighborhood $P\;=\;\{P_{1},P_{2},\ldots ,P_{N}\}\;\epsilon \;R^{K\times N\times (3+C)}$ of the point cloud as input. The encoder consists of five levels. At each level, the encoder sequentially performs the farthest point sampling and SFSG module. The encoder progressively compresses the point cloud size while expanding feature dimensions. Specifically, the first layer samples 8192 center points with a ball query radius of 1, and C1 is 32 and C2 is 64 in the SFSG module; the second layer samples 2048 points with a ball query radius of 2, and C1 is 64 and C2 is 128 in the SFSG module; the third layer samples 512 points with a ball query radius of 4, and C1 is 128 and C2 is 256 in the SFSG module; the fourth layer samples 128 points with a ball query radius of 8, and C1 is 256 and C2 is 512 in the SFSG module; and the fifth layer samples 32 points with a ball query radius of 16, and C1 is 512 and C2 is 1024 in the SFSG module. In each layer, the random selection number of neighboring points is set to 32.

In the decoder, the interpolation operation restores the size of the original point cloud by the distance-weighted average method [4] of the nearest three points, and the output feature of each interpolation operation is connected with the output feature of the corresponding encoder by the skip connection. The connection feature is input into the MLP layer. Finally, the fully connected layer is applied to classify.

Based on the SFSG module, SFSGNet can effectively handle irregular point clouds and describe the structure of point cloud objects. SFSGNet can achieve efficient and accurate point cloud semantic segmentation to assign a high-level semantic label for each point.

The MLP is adopted as the core operator for feature expansion in the SFSG module due to its strengths in adapting to irregular point clouds, enhancing feature expansion efficiency and enabling flexible cross-feature fusion. It can not only address the core challenges in processing airborne LiDAR point clouds but also circumvent the issues of high complexity and strong scene sensitivity associated with convolutional operators such as EdgeConv, KPConv and PAConv.

The MLP has a core limitation: it processes the features of individual points in an isolated manner and cannot explicitly model the spatial correlations between adjacent points. Unlike convolutional operators that inherently possess the ability to aggregate neighborhood information, the MLP is unable to directly capture geometric structural features of point clouds such as density variations, surface curvature and the relative positions of adjacent points. This drawback may lead to insufficient extraction of fine-grained local features, particularly for categories with distinct geometric characteristics in airborne point clouds, thus compromising the integrity of feature representation.

To compensate for the MLP’s limitation in explicitly capturing local geometric structures, the paper devises a synergistic design integrating ball-query-based neighborhood grouping, dual-path feature fusion, hierarchical multi-scale sampling and aggregated feature integration, which supplements the MLP with a comprehensive spatial information capture mechanism. Ultimately, this enables the SFSG module to generate highly expressive spatial structural features that fuse semantic, spatial and geometric information.

### 2.3. Pruned SFSGNet

To further improve the deployment efficiency of SFSGNet, we introduce channel pruning to remove redundant feature channels in the hierarchical encoder–decoder structure [35]. During pruning, sparsity regularization is imposed on channel scaling factors to identify less informative channels. After pruning, a narrower and more compact model is obtained, and the pruned SFSGNet is termed P-SFSGNet. As with most model compression methods, channel pruning may lead to a slight decrease in segmentation accuracy, especially for minority categories or geometrically confusing classes, because their discriminative cues are more sensitive to feature compression. Nevertheless, the pruned P-SFSGNet reduces the number of parameters, computational cost, and memory consumption while improving inference efficiency. Therefore, the full SFSGNet is more suitable for accuracy-oriented airborne LiDAR point cloud interpretation tasks, whereas P-SFSGNet is more suitable for latency-sensitive or resource-constrained deployment scenarios.

## 3. Experiment

### 3.1. Datasets

**Figure 3:**
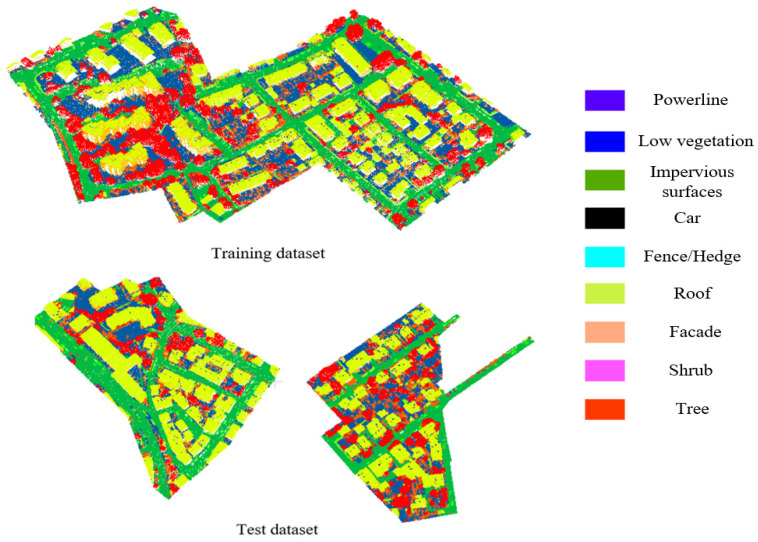
Distribution of points per category in the ISPRS benchmark dataset.

ISPRS 3D Labeling Benchmark Dataset: The performance of SFSGNet is evaluated on the ISPRS 3D Labeling Benchmark Dataset. The dataset is derived from airborne laser scanning (ALS) point cloud data collected in the Vaihingen area of Germany and built for semantic understanding tasks in urban scenes. It mainly covers high-density urban built-up areas, featuring residential land patterns and abundant vegetation elements, with notable structural complexity and ground object diversity. The dataset defines nine semantic categories in total, namely powerlines, low vegetation, impervious surfaces, cars, fence, roofs, facades, shrubs and trees. The distribution of points per category in the training and test dataset is shown in Figure 3.

**Figure 4:**
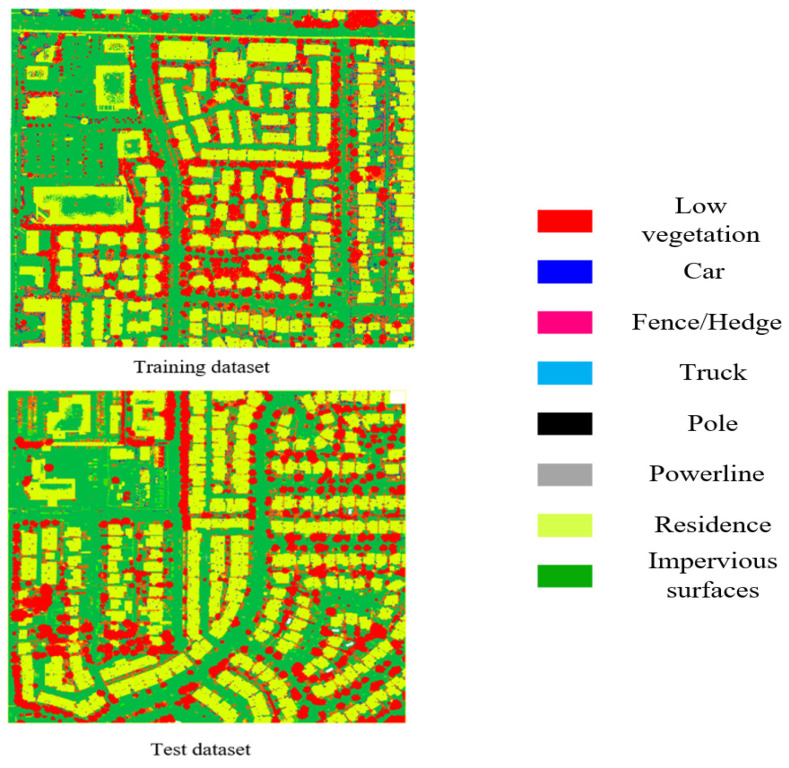
Distribution of points per category for the training set and test set of DALES dataset.

DALES dataset: To verify the generalization performance of the SFSGNet proposed in this paper, we further conduct experiments on the DALES dataset, which was released by the research team of the University of Dayton in 2020. This dataset provides a fine-grained semantic annotation system for complex urban scenes. The DALES dataset has eight classes: powerlines, low vegetation, impervious surfaces, cars, fences, residences, trucks, and poles. We use this label set in all DALES experiments. The selected regions are split into 29 regions for training and 11 regions for testing, and the class distributions of the training and test sets are also reported, as shown in Figure 4.

The OpenGF dataset is an ultra-large-scale airborne laser scanning dataset for ground filtering, constructed from open airborne laser scanning (ALS) point clouds collected in four countries worldwide [36]. It covers an area of approximately 47.7 square kilometers and contains more than 542 million labeled points. Different from conventional semantic segmentation benchmark datasets, OpenGF mainly focuses on ground extraction. Therefore, the dataset defines ground and non-ground as the annotation categories. Test 1 is the test dataset we employ, consisting of a challenging point cloud covering approximately 6.6 ${km}^{2}$. The region spans rural areas, small towns and mountainous terrain with diverse and complex topography. Test 1 has an average point density of about 6 points$/m^{2}$. All tests in this paper are conducted on Test 1.

### 3.2. Implementation Details

SFSGNet was implemented in TensorFlow and trained on a single GPU. The optimizer was Adam, with an initial learning rate of 0.005. An exponential decay schedule was adopted, where the decay step and decay rate were set to 2000 and 0.8, respectively. The maximum number of training epochs was 1000, and the batch size was set to 2. Each training sample contained 20,586 points. During training, several data augmentation strategies were applied, including random point dropout, random rotation around the z-axis, random scaling, Gaussian jittering, small-angle random rotation perturbation, and random shifting. For feature preprocessing, the XYZ coordinates of each scene were mean-centered. To alleviate the optimization bias caused by class imbalance, a category-weighted sparse softmax cross-entropy loss was employed during training. The loss is defined as

(9) $$L=-\frac{1}{N}\sum_{i=1}^{N}w_{y_{i}}\log p_{i,y_{i}}$$

where N represents the number of input points; $y_{i}$ represents the ground-truth label of the i-th point; $p_{i,y_{i}}$ represents the predicted probability of the correct class; and $w_{y_{i}}$ represents the class weight corresponding to label $y_{i}$. The class weight was computed according to the relative frequency of each class in the training set as

(10) $$w_{c}=\frac{1}{\log(1.2+p_{c})}$$

where $p_{c}$ represents the relative frequency of class c. This weighting strategy was used to reduce the dominance of high-frequency categories during optimization and to provide relatively stronger supervision for underrepresented categories. For all comparative experiments, the same dataset split and evaluation protocol were adopted to ensure fair comparison.

The effectiveness of the category-weighted loss is particularly reflected in the learning of small-sample categories. Since minority classes occupy a relatively small proportion of points in the training set, their contribution to the total loss would be limited under the standard cross-entropy formulation, which may cause the optimization process to be dominated by majority categories. The adopted weighting strategy increases the penalty for misclassified minority-class points, thereby enhancing their influence on parameter updates and improving the discriminative learning of underrepresented categories. However, the improvement brought by the weighted loss is still limited when the target categories also suffer from severe geometric ambiguity or unclear class boundaries.

### 3.3. Evaluation Criterion

To quantitatively evaluate the performance on 3D point cloud semantic segmentation tasks, the following key metrics are calculated: precision, recall, overall accuracy (OA), and mean F1 score. Overall accuracy is the most intuitive metric, representing the proportion of correctly classified points among all test points. The F1 score is the harmonic mean of precision and recall, which comprehensively considers the model’s precision and recall. The definitions of precision, recall, OA and F1 score are as follows:

(11) $$Precision=\frac{TP}{FP+TP}$$

(12) $$Recall=\frac{TP}{FN+TP}$$

(13) $$OA=\frac{TN+TP}{FP+TP+FN+TN}$$

(14) $$F1=2\times \frac{Recall\times Precision}{Recall+Precision}$$

In the experimental results, we independently count the number of point cloud samples correctly classified as true positives (TPs), true negatives (TNs), false positives (FPs), and false negatives (FNs).

In the quantitative evaluation of 3D point cloud semantic segmentation, per-category statistics define TPs as the number of points where both the ground-truth and model-predicted labels match the target category, FNs as the number of points that are the target category in the ground truth but predicted as other categories, FPs as the number of points that are non-target categories in the ground truth yet are predicted as the target category, and TNs as the number of points where neither the ground-truth nor predicted labels belong to the target category. OA is the ratio of the sum of TPs and TNs to the total number of samples, representing the global correct classification ratio of all points. The sum of these four point counts equals the total number of points in the test set, which also forms the basis for calculating the F1 score for each category.

The distribution characteristics of point cloud sample counts directly impact evaluation results. An imbalance in point cloud sample counts across categories, with dominant categories having far more points than minority ones, directly leads to low absolute TP values and poor F1 score performance for minority categories. For this reason, a category-weighted loss function designed based on the proportion of point samples in each category is adopted in this research to mitigate the model’s prediction bias toward dominant categories. Furthermore, the uneven spatial density of point clouds results in a higher proportion of FPs and FNs in sparse regions due to the insufficient number of point samples, which in turn affects the evaluation metrics of local segmentation. Overall, the number of point cloud samples not only determines the numerical scale of TPs, TNs, FPs and FNs and serves as the core foundation for calculating segmentation evaluation metrics but also acts as a key factor causing relevant evaluation issues such as category imbalance and uneven spatial density.

### 3.4. Experiment on ISPRS 3D Labeling Benchmark Dataset

The evaluation metrics of SFSGNet on the ISPRS benchmark dataset are shown in Table 1.

According to the F1 score in Table 1, SFSGNet achieves good segmentation performance on the powerline, low vegetation, impervious surfaces, car, roof, facade and tree categories. However, due to shrub and fence categories lacking clear boundaries between them, SFSGNet demonstrates poor performance on these two categories.

**Figure 5:**
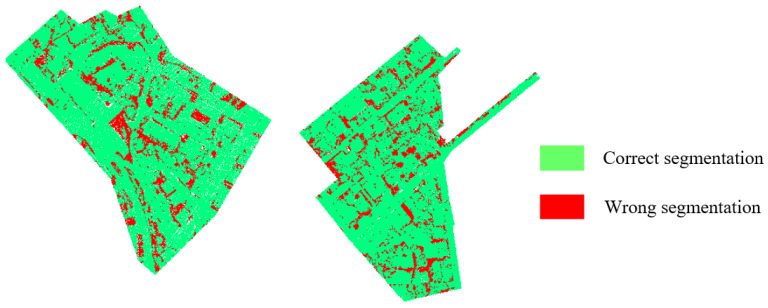
Comparison between the semantic segmentation result and ground truth on the ISPRS test dataset.

**Figure 6:**
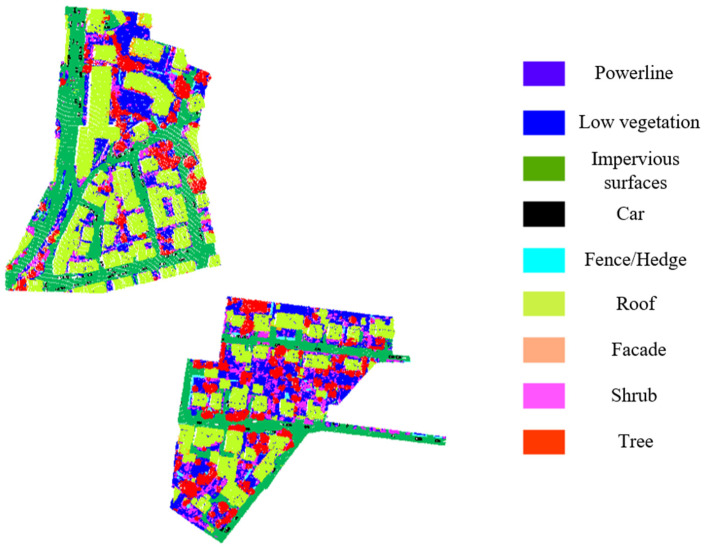
Semantic segmentation results of SFSGNet on the ISPRS test dataset.

To verify the effectiveness of the proposed SFSGNet, this paper selects several mainstream models for comparison, including WhuY3 [37], WhuY4 [38], PointCNN [6], PointNet++ [4], GACNet [39], D-FCN [40], MCFN [41], KPConv [5], and GACNN [42]. Table 2 presents the F1 scores for each class, the average F1 score, and the overall accuracy for all these models. In Table 2, bold values indicate the best performance among the ten models, and underlined values indicate the second-best performance. Figure 5 shows a comparison of classification results on the Vaihingen test set, where green points represent correctly classified samples and red points represent misclassified ones. Figure 6 displays the classification results of SFSGNet on the test set.

As shown in Table 2, SFSGNet achieves the best F1 score on the low vegetation, car and roof categories. SFSGNet and MCFN both achieve the best performance in terms of average F1 score. SFSGNet achieves the best performance in terms of OA.

Compared with the other nine methods, SFSGNet achieves the best performance in terms of average F1 score and OA. Our SFSGNet exhibits excellent segmentation performance on the ISPRS dataset.

The evaluation results on the ISPRS dataset demonstrate that SFSGNet achieves strong overall segmentation performance, especially on dominant categories such as low vegetation, cars, roofs, and several other major classes. However, some minority and confusing categories still remain challenging. On this dataset, the fence and shrub categories obtain relatively low F1 scores of 54.3% and 53.5%, respectively. To alleviate the optimization bias caused by class imbalance, a category-weighted cross-entropy loss is introduced during training, which further improves the overall accuracy and mean F1 score of SFSGNet by strengthening the supervision of underrepresented categories. Nevertheless, the performance of fences and shrubs remains lower than that of dominant classes, indicating that their limitations are not solely caused by insufficient sample proportions. Specifically, fence points are usually sparse, thin, and discontinuous, making them easily affected by neighboring facade-like structures, while shrub points often share similar height distribution and irregular geometric characteristics with low vegetation, which increases the difficulty of discrimination based on local structural features.

### 3.5. Experiments on DALES Dataset

**Figure 7:**
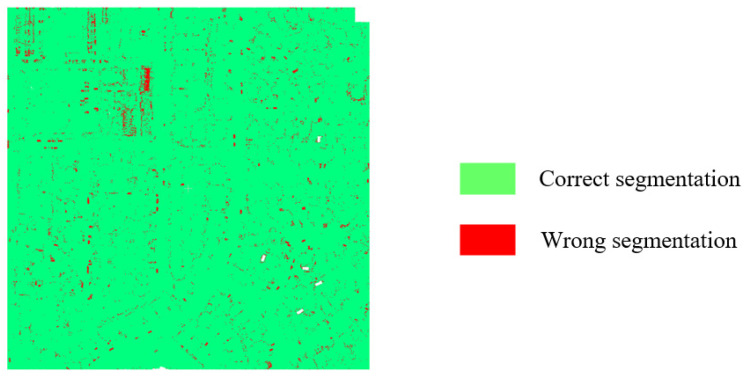
Comparison between the semantic segmentation result and ground truth on the DALES test dataset.

To further verify the effectiveness of the proposed SFSGNet on larger-scale and more complex urban scene point clouds, semantic segmentation experiments are conducted on the DALES dataset. Figure 7 compares the segmentation results with ground-truth labels, where green points indicate correctly segmented instances and red points indicate errors.

This experiment compares the proposed SFSGNet with nine mainstream models: WhuY3, WhuY4, PointNet++, GACNet, PointCNN, D-FCN, MCFN, GACNN, and KPConv. The per-class F1 scores, mean F1 score, and overall accuracy for all these models are listed in Table 3. In this table, bold values indicate the best performance among the ten models, and underlined values indicate the second-best performance. As shown in Table 3, SFSGNet achieves the highest mean F1 score and overall accuracy among all ten models. Notably, SFSGNet achieves the best F1 scores on the powerline, low vegetation, impervious surfaces, fence, residence and truck categories, which shows that its dual-path design is particularly adept at capturing the complex structures.

These comparisons and analyses demonstrate that SFSGNet delivers superior semantic segmentation performance on the large-scale DALES dataset. Our SFSGNet has strong generalization capability.

On the DALES dataset, SFSGNet also presents strong overall segmentation performance on dominant classes, while the truck category achieves an F1 score of only 63.6%. Although the adopted category-weighted cross-entropy loss effectively alleviates class imbalance and enhances overall performance, the truck category still shows limited improvement. This is because truck instances usually occupy a small proportion of points and often contain insufficient geometric details in airborne LiDAR scenes, and their local structures may also be similar to cars or nearby artificial objects. Therefore, even though the weighted loss mitigates the impact of class imbalance, it cannot completely eliminate the intrinsic difficulty of distinguishing geometrically confusing minority categories such as trucks.

### 3.6. Experiments on OpenGF Dataset

This experiment compares the proposed SFSGNet with other methods on the OpenGF dataset. The per-class F1 scores, mean F1 score, and overall accuracy of all methods are listed in Table 4. In this table, bold values indicate the best performance among all compared methods, and underlined values indicate the second-best performance. As shown in Table 4, SFSGNet achieves the highest mean F1 score and overall accuracy.

These comparisons and analyses demonstrate that SFSGNet delivers superior classification performance on the ground points and non-ground points. Our SFSGNet exhibits strong generalization capability across large-scale airborne LiDAR scenes with diverse mountainous terrain conditions.

### 3.7. Ablation Experiment and Analysis

To comprehensively validate the effectiveness of each key component and the rationality of hyperparameter settings in the proposed method, we construct twelve ablation models and one baseline model, totaling nine models for controlled comparison. The detailed settings are as follows:

Model 1: SFSGNet without SFSG module. The entire spatial feature structure generation (SFSG) module is removed, while the encoder–decoder, sampling, and interpolation pipelines remain unchanged.

Model 2: SFSGNet with AFE block, without CFE block, CFEF block, CF block, DFA block. Only the attribute feature extension (AFE) block is retained, and other blocks are removed.

Model 3: SFSGNet with CFE block, without AFE block, CFEF block, CF block, DFA block. Only the coordinate feature expansion block is retained, and other blocks are removed.

Model 4: SFSGNet with AFE block and CFE block, without CFEF block, CF block, DFA block. Only the attribute feature extension (AFE) block and the coordinate feature expansion (CFE) block are retained, and other blocks are removed.

Model 5: SFSGNet with CFEF block and CF block, without DFA block. Only the CFEF block and CF block are retained, and another fusion is removed.

SFSGNet: The complete proposed network with default settings, including the SFSG module.

SFSGNet on ISPRS dataset without intensity: The entire structure of SFSGNet is retained, but the input data only has three coordinates (x,y,z), and the reflection intensity is removed.

Model 7: To investigate the sensitivity of the model to the ball-query radii, all original network structures are retained, and only the ball-query radii are scaled up by a factor of two. Specifically, the original ball-query radii set {1, 2, 4, 8, 16} is adjusted to {2, 4, 8, 16, 32}.

Model 8: To investigate the sensitivity of the model to the ball-query radii, all original network structures are retained, and only the ball-query radii are scaled down by a factor of two. Specifically, the original ball-query radii set {1, 2, 4, 8, 16} is adjusted to {0.5, 1, 2, 4, 8}.

Model 9: To investigate the sensitivity of the model to the channel, the feature channel dimensions C1 and C2 in each layer are doubled. The original {(32, 64), (64, 128), (128, 256), (256, 512), (512, 1024)} is changed to {(64, 128), (128, 256), (256, 512), (512, 1024), (1024, 2048)}. All other parameters remain the same as before.

Model 10: To investigate the sensitivity of the model to the channel, the feature channel dimensions C1 and C2 in each layer are scaled down by a factor of two. Specifically, the original feature channel dimension pairs {(32, 64), (64, 128), (128, 256), (256, 512), (512, 1024)} are adjusted to {(16, 32), (32, 64), (64, 128), (128, 256), (256, 512)}. All other parameters remain the same as before.

Model 11: With the other configurations of the model remaining unchanged, the number of neighboring points is set to 64.

Model 12: With the other configurations of the model remaining unchanged, the number of neighboring points is set to 16.

As shown in Table 5, the full SFSGNet outperforms the variant without the SFSG module on both OA and F1 score. This improvement demonstrates the effectiveness of the proposed SFSG module. By enabling more comprehensive interactions between attribute features and coordinate features, the SFSG module can generate highly expressive spatial structure features in both semantic and spatial contexts, thereby significantly improving semantic segmentation performance.

In addition, Table 6 presents the model complexity of SFSGNet without the SFSG module, P-SFSGNet, and the full SFSGNet. The parameters and GFLOPs are related to the model structure. The parameters are derived by summing the elements of all trainable variable tensors. The GPU memory is measured during the model training process. The inference points/second is calculated over the total inference time, including neighborhood construction and model inference.

Compared with SFSGNet without the SFSG module, the full SFSGNet increases the number of parameters, GFLOPs, and GPU memory by 19%, 33%, and 25%, respectively, while reducing the inference throughput by 4.7%, which indicates that the introduction of the SFSG module increases network complexity. The pruned variant P-SFSGNet provides an intermediate trade-off. Specifically, compared with SFSGNet without the SFSG module, P-SFSGNet increases the number of parameters, GFLOPs, and GPU memory by 7.6%, 17.6%, and 15.8%, respectively, while decreasing the inference throughput by only 2.1%. Compared with the full SFSGNet, P-SFSGNet reduces the number of parameters, GFLOPs, and GPU memory by 9.4%, 11.8%, and 7.6%, respectively, and improves the inference throughput by 2.8%. Therefore, although the full SFSGNet achieves the strongest representation capability, P-SFSGNet offers a more favorable balance between computational cost, hardware consumption, and inference efficiency.

## 4. Discussion

Extensive experiments on the ISPRS 3D Labeling Benchmark, DALES, and OpenGF datasets validate the superior performance of SFSGNet. On the ISPRS dataset, SFSGNet achieves an average F1 score of 74.4% and an overall accuracy (OA) of 85.0%, both ranking first among all compared methods. It also obtains the highest F1 scores on low vegetation, cars and roofs and outperforms the other nine state-of-the-art approaches. On the DALES dataset, SFSGNet delivers an average F1 score of 89.3% and an OA of 98.1%, surpassing all nine competing methods, with the best F1 score performance on powerlines, low vegetation, impervious surfaces, fences, residences and trucks, and on the OpenGF dataset, SFSGNet achieves an average F1 score of 98.2% and an overall accuracy (OA) of 98.5%.

Efficiency analysis shows that, compared with the model without the SFSG module, the full SFSGNet increases parameters by 19%, GFLOPs by 33% and GPU memory usage by 25%, while inference throughput only decreases by 4.7%. Compared with the full SFSGNet, P-SFSGNet reduces parameters by 9.4%, GFLOPs by 11.8% and GPU memory usage by 7.6%, while inference throughput is increased by 2.8%. P-SFSGNet achieves a well-balanced trade-off between performance and resource consumption, but the full SFSGNet achieves the optimal segmentation performance.

The dual-path feature aggregation block effectively integrates geometric coordinates and attribute features to generate expressive spatial structure features, addressing the irregularity of point clouds and insufficient feature interaction. The strong results of the three benchmark tests confirm that SFSGNet has strong generalization ability in different urban scenarios, providing reliable support for 3D city modeling, autonomous driving maps, and infrastructure monitoring.

Future work will explore the application of SFSGNet in weakly supervised learning. Recent advances in weakly supervised classification learning include the proposal of Point-SAM by Zhou et al. [43], which adapts the Segment Anything Model (SAM) to 3D point clouds for flexible interactive segmentation through prompt engineering, as well as the investigation by Yu et al. [44] on weakly supervised point cloud segmentation strategies, demonstrating that point-level annotations can greatly reduce labeling costs while maintaining competitive performance. Inspired by these methodologies, particularly pseudo-label learning and label propagation techniques, we aim to integrate the spatial feature structure generation mechanism of SFSGNet into weakly supervised frameworks. This integration seeks to reduce the dependence on dense manual annotations and thereby enhance the model’s generalization and adaptability to large-scale, low-label airborne LiDAR data.

In practical applications, airborne LiDAR point clouds usually have a large volume and require compression for efficient storage and transmission. However, previous studies [45,46] have demonstrated that LiDAR point cloud compression may induce information loss, which distorts local geometric structures and point spatial distributions and further degrades the performance of perception tasks, such as semantic segmentation. The proposed SFSGNet is designed to achieve favorable segmentation performance on raw point clouds. When point cloud compression is applied to the training and test datasets, the loss of geometric structural information will lead to a certain drop in accuracy of SFSGNet. Accordingly, enhancing the overall capability and robustness of SFSGNet for compressed point clouds remains a critical direction for future research.

## 5. Conclusions

This paper presents SFSGNet, a highly effective spatial feature structure generation network dedicated to airborne LiDAR point cloud semantic segmentation. The core innovation lies in the well-designed SFSG module, which adopts a dual-path heterogeneous fusion architecture to deeply integrate geometric coordinate features and attribute features, generating highly expressive spatial structure features. This design excellently addresses the challenges of irregular point cloud distribution and insufficient interaction between spatial and semantic features, enabling the network to accurately capture fine-grained object structures and complex contextual information in urban scenes.

Extensive experimental results on three authoritative benchmark datasets, ISPRS 3D Labeling Benchmark, DALES and OpenGF, fully demonstrate that SFSGNet achieves state-of-the-art segmentation performance with outstanding overall accuracy and mean F1 score, surpassing nine mainstream comparison methods comprehensively. Meanwhile, the model maintains a favorable trade-off between segmentation precision and computational efficiency, showing strong stability and generalization capability across different scene layouts, point densities and semantic categories.

The proposed SFSGNet not only advances the technical research of 3D point cloud semantic segmentation but also provides reliable and efficient technical support for realistic applications, including large-scale 3D city modeling, intelligent infrastructure monitoring, high-precision mapping for autonomous driving and urban digital twin construction. With excellent performance and strong practicality, SFSGNet represents a high-quality solution for airborne LiDAR point cloud understanding and has broad application prospects in the field of 3D spatial information processing.

## Figures and Tables

**Table 1:** Per-category precision, recall and F1 score of SFSGNet on the ISPRS benchmark dataset.

Evaluation Criterion	Pow	Low_v	Imp_s	Car	Fence	Roof	Facade	Shrub	Tree
Precision	68.3	85.5	91	86.8	54.8	94.6	71.6	44.5	80.9
Recall	65.3	81.0	93.4	75.5	35.3	95.0	53.1	53.1	85.7
F1	66.8	83.2	92.2	80.7	43.0	94.8	61.0	48.4	83.2

**Table 2:** Per-category F1 score, average F1 score, and OA of SFSGNet and other models on the ISPRS benchmark dataset.

Categories	F1 Score (%)									Average F1 Score (%)	OA (%)
	Pow	Low_v	Imp_s	Car	Fence	Roof	Facade	Shrub	Tree		
WhuY3 [37]	37.1	81.4	90.1	63.4	23.9	93.4	47.5	39.9	78.0	61.6	82.3
WhuY4 [38]	42.5	82.7	91.4	74.7	53.7	94.3	53.1	47.9	82.8	69.2	84.9
PointCNN [6]	61.5	82.7	91.8	75.8	35.9	92.7	57.8	49.1	78.1	69.5	83.3
PointNet++ [4]	50.4	82.2	91.0	68.0	28.4	91.8	49.8	37.8	78.5	64.2	82.3
GACNet [39]	47.0	82.7	91.4	72.3	31.1	92.6	56.0	44.7	79.5	66.4	82.8
D-FCN [40]	70.4	80.2	91.4	78.1	37.0	93.0	60.5	46.0	79.4	70.7	82.2
MCFN [41]	74.5	82.3	91.8	79.0	37.5	94.7	**61.7**	48.7	**83.3**	72.6	84.4
KPConv [5]	47.3	82.3	91.4	77.2	32.8	93.9	61.4	45.4	81.2	68.1	83.7
GACNN [42]	**76.0**	81.8	**93.0**	77.7	37.8	93.1	58.9	46.7	78.9	71.5	83.2
PointSSM [19]	68.9	82.8	91.9	78.9	42.1	94.2	60.8	48.8	81.5	72.2	83.8
Att-AdaptNet [31]	71.2	83.1	92.7	79.2	48.6	94.5	61.3	50.3	82.9	73.6	84.7
P-SFSGNet	63.4	83.0	92.0	78.3	48.2	94.4	60.1	52.6	82.6	72.7	84.8
SFSGNet	66.8	**83.2**	92.2	**80.7**	**54.3**	**94.8**	61.0	**53.5**	83.2	**74.4**	**85.4**

Bold values indicate the best and underlined values indicate the second-best.

**Table 3:** Per-category F1 score, average F1 score and overall accuracy of SFSGNet and other models on the DALES dataset.

Categories	F1 Score (%)								Average F1 Score (%)	OA (%)
	Pow	Low_v	Imp_s	Car	Fence	Res	Truck	Pole		
WhuY3 [37]	35.7	91.5	98.1	61.7	45.8	94.5	31.6	37.7	62.1	95.9
WhuY4 [38]	43.7	92.4	97.7	73.3	56.8	95.2	42.6	45.0	68.3	96.0
PointCNN [6]	42.1	95.7	98.2	57.8	68.9	97.8	9.20	73.1	67.9	97.2
PointNet++ [4]	88.8	95.4	97.0	86.0	63.2	94.2	46.5	57.1	78.5	95.7
GACNet [39]	57.2	92.4	96.8	87.7	64.2	96.3	49.3	46.7	73.8	96.8
D-FCN [40]	69.4	90.8	96.9	86.1	66.8	95.6	53.4	58.5	77.2	97.1
MCFN [41]	73.3	93.7	97.0	89.2	67.1	95.4	58.2	67.2	80.1	97.4
KPConv [5]	97.7	97.0	98.5	92.1	77.7	98.3	59.1	**85.7**	88.3	97.8
GACNN [42]	97.8	96.9	**98.6**	92.5	77.4	98.4	61.4	85.1	87.0	97.6
Att-AdaptNet [31]	97.6	96.8	98.4	91.4	79.8	96.5	60.7	84.4	88.2	97.7
PointSSM [19]	**98.6**	96.8	98.5	93.1	80.2	98.6	61.6	85.3	89.1	97.8
P-SFSGNet	97.1	96.4	97.5	91.5	78.0	97.2	62.6	84.7	88.1	98.0
SFSGNet	98.5	**97.2**	**98.6**	**93.2**	**80.3**	**98.7**	**62.7**	85.4	**89.3**	**98.1**

Bold values indicate the best and underlined values indicate the second-best.

**Table 4:** Per-category F1 score, average F1 score, and OA of SFSGNet and other models on the the OpenGF dataset.

Categories	F1 Score (%)		Average F1 Score (%)	OA (%)
	Ground Points	Non-Ground Points		
WhuY3 [37]	92.0	92.6	92.3	84.7
WhuY4 [38]	93.5	93.7	93.6	84.9
PointCNN [6]	93.3	93.8	93.6	85.0
PointNet++ [4]	97.3	97.8	97.6	86.4
GACNet [39]	**97.6**	97.9	97.8	91.1
D-FCN [40]	93.6	94.5	94.1	85.2
MCFN [41]	94.1	94.5	94.3	85.7
KPConv [5]	97.5	98.0	97.8	91.7
GACNN [42]	95.2	95.4	95.3	86.4
PointSSM [19]	95.8	96.2	96.0	87.6
Att-AdaptNet [31]	96.3	97.5	96.9	88.9
P-SFSGNet	97.4	97.7	97.6	90.4
SFSGNet	**97.6**	**98.1**	**97.9**	**91.9**

Bold values indicate the best and underlined values indicate the second-best.

**Table 5:** Ablation experiment on the ISPRS test dataset.

Model	F1 Score (%)									Average F1 Score (%)	OA (%)
	Pow	Low_v	Imp_s	Car	Fence	Roof	Facade	Shrub	Tree		
Model 1	48.1	81.9	89.1	67.7	29.7	92.8	49.1	39.1	78.1	64.0	82.3
Model 2	48.3	82.3	89.3	73.4	46.2	93.3	51.9	50.1	81.5	68.5	82.6
Model 3	49.5	82.9	89.8	73.8	46.8	93.4	53.1	50.5	81.9	69.1	82.8
Model 4	51.4	83.0	91.4	79.6	50.7	94.1	58.3	52.4	82.6	71.5	84.2
Model 5	66.2	82.4	92.0	79.4	53.9	94.5	59.3	53.2	82.6	73.7	84.8
SFSGNet	**66.8**	**83.2**	**92.2**	**80.7**	**54.3**	**94.8**	**61.0**	**53.5**	**83.2**	**74.4**	**85.0**
SFSGNet on ISPRS dataset without intensity	61.4	82.3	91.3	74.7	48.4	94.0	57.8	49.0	81.7	71.2	83.2
Model 7	62.7	82.9	91.8	77.3	50.9	94.3	60.1	52.6	82.3	72.8	84.6
Model 8	60.1	83.2	90.8	78.8	51.2	93.3	57.4	52.0	81.8	72.0	84.3
Model 9	65.7	82.1	91.9	77.9	52.5	94.4	60.9	53.4	82.5	73.4	84.9
Model 10	65.5	82.0	91.7	78.9	52.2	93.8	60.2	52.4	82.5	73.2	84.5
Model 11	66.6	83.1	92.0	80.5	54.1	94.6	60.7	53.3	83.0	74.2	84.8
Model 12	50.2	82.0	89.9	74.4	49.8	93.1	51.5	50.8	79.5	69.0	83.7

Bold values indicate the best and underlined values indicate the second-best.

**Table 6:** Complexity of the network.

Methods	Parameters	GFLOPs	GPU Memory	Inference Points/Second
SFSGNet without SFSG module	1.416 M	32.71	4.17 GB	34,126
P-SFSGNet	1.524 M	38.48	4.83 GB	33,415
SFSGNet	1.682 M	43.62	5.23 GB	32,519

## Data Availability

The original contributions presented in this study are included in the article. Further inquiries can be directed to the corresponding author.
